# Identification of Hub lncRNAs Along With lncRNA-miRNA-mRNA Network for Effective Diagnosis and Prognosis of Papillary Thyroid Cancer

**DOI:** 10.3389/fphar.2021.748867

**Published:** 2021-10-13

**Authors:** Haiyan Li, Feng Liu, Xiaoyang Wang, Menglong Li, Zhihui Li, Yongmei Xie, Yanzhi Guo

**Affiliations:** ^1^ College of Chemistry, Sichuan University, Chengdu, China; ^2^ Department of Thyroid Surgery, West China Hospital of Sichuan University, Chengdu, China; ^3^ Laboratory of Thyroid and Parathyroid Disease, Frontiers Science Center for Disease-Related Molecular Network, West China Hospital of Sichuan University, Chengdu, China

**Keywords:** papillary thyroid cancer, hub lncRNAs, lncRNA-miRNA-mRNA ceRNA network, diagnosis, prognosis signature, bioactive chemicals

## Abstract

Long noncoding RNAs (lncRNAs) play important roles in tumorigenesis and progression of different cancers and they have been potential biomarkers for cancer diagnosis and prognosis. As the most common endocrine malignancy, precise diagnosis and prognosis of papillary thyroid cancer (PTC) is of great clinical significance. Here, we aim to identify new hub lncRNAs for marking PTC and constructed prognostics signatures based on lncRNA- miRNA-mRNA competing endogenous RNAs (ceRNA) network to predict overall survival (OS) and disease-free survival (DFS) respectively. Five reliable hub lncRNAs were identified by integrating differential genes of four Gene Expression Omnibus (GEO) gene chips using the RobustRankAggreg (RRA) method. Based on differential analyses and interaction prediction, a lncRNA-mRNA co-expression network and a lncRNA-miRNA-mRNA ceRNA network were established. Then a comprehensive function characterization of the five hub lncRNAs was performed, including validation dataset testing, receiver operating characteristic (ROC) curve analysis, and functional analysis on two networks. All results suggest that these five hub lncRNAs could be potential biomarkers for marking PTC. The ceRNA network was used to identify RNAs which were associated with PTC prognosis. Two prognostic signatures were developed using univariate and step-wise multivariate Cox regression analyses and both of them were independent prognostic indicators for PTC OS and DFS. Tumor microenvironment difference analysis between high and low-risk patients showed that dendritic cells activated and macrophages M0 may be a possible target for immunotherapy of PTC. In addition, disclosing the potential drugs that may reverse the expression of hub genes may improve the prognosis of patients with PTC. Here, connectivity map (CMap) analysis indicates that three bioactive chemicals (pioglitazone, benserazide, and SB-203580) are promising therapeutic agents for PTC. So, the paper presents a comprehensive study on diagnosis, prognosis, and potential drug screening for PTC based on the five hub lncRNAs identified by us.

## Introduction

Thyroid cancer is the most common endocrine malignancy and the incidence has been rapidly increasing in the past 4 decades (Murugan et al., 2018). As the most common histological type of thyroid cancer, papillary thyroid cancer (PTC) accounts for approximately 85% of all cases (Fagin and Wells, 2016; Kitahara and Sosa, 2016). The incidence of PTC has also been increasing rapidly in most countries (La Vecchia et al., 2015). Generally it has an excellent prognosis but the recurrence to distant organs always threaten the patient’s life (Ito et al., 2010). In the last few years, research has been performed to promote our understanding of molecular mechanisms of PTC (Nikiforov and Nikiforova, 2011; Fagin and Wells, 2016). Studies have suggested the crucial roles of lncRNAs, circRNAs, miRNAs, and mRNAs in PTC’s diagnosis and prognosis (Chen et al., 2019; Huang et al., 2020; Xu and Jing, 2021). Discovering more biomarker genes and developing reliable prognostic signatures could remarkably promote the development of clinical treatment.

As we all know, human cancers are frequently correlated with the change of transcription pattern and the transcriptome is not only restricted to protein-coding RNAs but also refers to the multiple noncoding members (Liz and Esteller, 2016). The biological roles of RNAs in tumorigenesis and progression has become an interesting research hotpot. As the fundamental transcription regulators, lncRNAs could affect cellular functions including apoptosis, cycle regulation, proliferation, migration, and invasion by regulating expressions of many salient genes (Fang and Fullwood, 2016). Nowadays, competing endogenous RNAs (ceRNAs) have been proven to play a prominent role in cancer initiation and progression and might be explored as diagnostic markers or therapeutic targets (Qi et al., 2015).

Meanwhile, cancer biomarkers need to have a strong specificity for a particular disease condition and lncRNAs have been emerging as crucial players in the control of gene expression (Iaccarino and Klapper, 2021). Previous studies have shown the marked heterogeneity in lncRNA expression between individual cancer cells so that lncRNA have a much higher cell/tissue specificity of expression in comparison to other ncRNAs and mRNAs (Silva et al., 2015; Wu et al., 2021). Besides, lncRNAs are often stable in clinical samples and can easily be detected by common techniques, such as quantitative real-time PCR, sequencing, and microarray hybridization (Silva et al., 2015). The patterns of lncRNAs deregulation in primary tumor tissues have been found in bodily fluids, including plasma and urine (Silva et al., 2015), which presents an opportunity to develop lncRNA-based biomarker tools that are convenient, minimally invasive, and may be easily accepted by patients.

Studies have indicated that lncRNAs could play important roles as ceRNAs in certain cancers, such as breast cancer, colorectal cancer, pancreatic cancer, and so on (Liu et al., 2021; Rong et al., 2021; Zeng et al., 2021). They also could exert carcinogenic effects as ceRNAs in PTC. For example, Sui et al. have revealed that, as a ceRNA of miR-214-3p, small nucleolar RNA host gene 3 (SNHG3) is an oncogenic lncRNA in PTC by binding with miR-214-3p to regulate the expression of proteasome 26S subunit non-ATPase 10 (PSMD10) (Sui et al., 2020). Further, Zhang et al. have proven that the lncRNA of FOXD2-AS1 is highly up-regulated in PTC and acts as a ceRNA to promote the expression of KLK7 by sponging miR-485-5p, resulting in cell proliferation and migration (Zhang et al., 2019). Moreover, the expression levels of lncRNAs and miRNAs may be directly associated with the good/bad prognosis and could be involved in carcinogenic or tumor-suppressive pathways, which mark them as potential prognostic biomarkers (Murugan et al., 2018; Hanna et al., 2019). For example, Chen et al. identified lncRNA TTTY10 as prognostic markers for predicting tumor recurrence in PTC (Chen et al., 2019). Zhao et al. screened out three lncRNAs of LINC00284, RBMS3-AS1, and ZFX-AS1 by constructing lncRNA-miRNA-mRNA network, which were found to be associated with PTC progression and prognosis (Zhao et al., 2018). Recently, Sun et al. found five lncRNAs which were associated with PTC patient survival time but only based on one individual GEO data set (Sun et al., 2020). However, potential lncRNA biomarkers which are more reliable and convincing are yet to be found, because the existing studies always give different crucial lncRNAs based on different individual databases. Until now, the field still lacks integration of different databases for a comprehensive validation on PTC hub lncRNA genes and the regulation characteristics of them are not well revealed.

In this study, we integrated the data from four GEO databases with the most PTC samples and the Cancer Genome Atlas (TCGA) so as to screen crucial lncRNAs. Five hub lncRNAs were achieved by robust rank aggregation (RRA) method for data integration of different databases. To comprehensively validate five hub genes, their expression difference analysis and the receiver operating characteristic (ROC) diagnostic analysis were performed based on four GEO datasets, TCGA and Gene Expression Profiling Interactive Analysis (GEPIA) database, respectively. Meanwhile, lncRNA-mRNA co-expression network and lncRNA-miRNA-mRNA ceRNA network were also constructed. Functional analysis on mRNAs involved in the two networks along with the deep-literature exploring five hub lncRNAs and these mRNAs all indicate that they are all involved in cancer-related functions. So, the five hub lncRNAs give promising potentiality for diagnosing PTC.

We also established two prognostic risk models for PTC OS and DFS, namely PTC-mi1m4 and PTC-m3, respectively, by screening all genes in ceRNA network. To elucidate the potential pathogenesis of PTC, Gene Oncology (GO), Kyoto Encyclopedia of Genes and Genomes (KEGG), and Disease Ontology (DO) enrichment analyses were performed. The proportions of 22 immune cells of PTC were analyzed to estimate the tumor microenvironment in patients with PTC. Among them, two immune cells were demonstrated to be associated with the prognosis of PTC and they may be the potential target of immunotherapy.

Finally, connectivity map (CMap) analysis was performed based on five prognosis-related mRNAs to screen potential bioactive chemicals. Three promising drugs were predicted as compounds that play vital roles in PTC-related biological processes and may provide potential treatment of PTC.

## Materials and Methods

### Data Collection and Pre-Processing

In this study, based on the same sequencing platform of Affymetrix Human Genome U133 Plus 2.0 Array from GEO database (http://www.ncbi.nlm.nih.gov/geo/query/acc.cdi), four gene chips with the most sample pairs were selected, including GSE29265 with 20 pairs of normal and PTC samples, GSE3678 with seven pairs, GSE3467 with nine pairs, and GSE33630 with 49 PTC samples and 45 normal samples. Furthermore, the RNA-Seq counts data of 501 PTC and 58 normal tissues were downloaded from TCGA data center (http://portal.gdc.cancer.gov/). Meanwhile, we obtained the clinical information of 496 PTC patients from cBioPortal for Cancer Genomics (http://www.cbioportal.org). After deleting PTC samples without either expression data or clinical information, 490 eligible PTC, and 58 normal tissues remained for the construction of PTC OS prediction model. Since 14 of 490 PTC samples lack clinical information about PTC DFS, the remaining 476 samples were used for DFS prediction.

In order to obtain lncRNA expression data, based on four GEO datasets, we only extracted genes annotated as “3prime_overlapping_ncRNA,” “antisense,” “sense_intronic,” “sense_overlapping,” “macro_lncRNA,” “lincRNA,” “non_coding,” “bidirectional_promoter_lncRNA,” and “misc_RNA.” After deleting genes with no expression in more than four samples, in total 1038 lncRNAs remained from four gene chips. In addition, 743 miRNAs and 16160 mRNAs were achieved from TCGA. Finally, all the raw data from GEO were normalized by the Normalize Between Arrays method in R package “limma” and those from TCGA were normalized by Trimmed Mean of M values (TMM) in R package “edgeR.”

### Differential Expression Analysis and RobustRankAggreg Method

Firstly, each GEO dataset was normalized using the normalize Between Arrays function in R package “limma.” Then, differential expression analysis was conducted on lncRNA expression data of four GEO individual datasets respectively also by R package “limma.” Here, considering the limited number of lncRNAs in GEO datasets, those with |logFC|>1 and adjusted *p* < 0.05 were selected as differentially expressed ones. However, different differential lncRNAs were extracted from different gene chips respectively. Here, in order to achieve more valid and representative differential lncRNAs as well as to remove the bath effect, RRA method in R package was employed to integrate the differentially expressed gene lists resulting from differential expression analysis of four individual datasets. The RRA method can detect genes that are ranked consistently better than expected and then assign a significance score for each gene. The significance scores provide a rigorous way to keep only the statistically relevant genes in the final list so that genes identified by this method will be robust, convincing, and significant (Kolde et al., 2012). Then, the significant differentially expressed lncRNAs selected by RRA method were considered as hub lncRNAs for further analysis.

Differential expression analysis with miRNA and mRNA expression data of TCGA database was performed using R software package “egdeR” with |logFC|>1 and adjusted *p* < 0.05. Finally, the “ggplot2” package was used to make the volcano plot visualized, revealing the distributions of all differential genes.

### Construction of lncRNA-mRNA Co-Expression Network

To establish the lncRNA-mRNA co-expression network, the Pearson correlation analysis was performed between expression levels of hub lncRNAs and differential mRNAs in TCGA so as to select co-expressed mRNAs that are correlated with hub lncRNAs with the coefficient value of |Cor|>0.5 and *p* < 0.05. The network graph of lncRNA-mRNA co-expression network was built and visualized by Cytoscape (Version:3.7.1, https://cytoscape.org/).

### Establishment of a lncRNA-miRNA-mRNA Network

For the purpose of constructing lncRNA-miRNA-mRNA ceRNA network, starBase v2.0 (http://starbase.sysu.edu.cn) was used to predict lncRNA-miRNA interactions. Those predicted miRNAs only proved to be differentially expressed by TCGA data are regarded as those which were used to construct the ceRNA network. mRNAs targeted by those miRNAs interacting with hub lncRNAs were predicted using miRTarbase (http://mirtarbase.cuhk.edu.cn/), miRDB (http://www.mirdb.org/), and TargetScan 7.2 (http://www.targetscan.org/vert_72/). Similarly, only those predicted target mRNAs that also differentially expressed TCGA can be involved in the ceRNA network. Finally, the lncRNA-miRNA-mRNA ceRNA network was established and visualized using Cytoscape (Version:3.7.1, https://cytoscape.org/).

### Functional Analysis

To characterize the function of mRNAs in lncRNA-mRNA co-expression network and those in lncRNA-miRNA-mRNA ceRNA network respectively, GO, KEGG, and DO enrichment analyses were all performed with “clusterProfiler” package for investigating biological process, molecular function, pathways, and related diseases.

### Development of Survival Signatures and Survival Analysis

Univariate Cox proportional hazards regression analysis was performed on all genes in lncRNA-miRNA-mRNA ceRNA network with *p* < 0.05 as the threshold to respectively identify OS-associated or DFS-associated lncRNAs, miRNAs, or mRNAs. Then, these genes were entered into the step-wise multivariate Cox regression analysis using R package “survminer” to screen out the key RNAs with great prognostic values. Finally, those RNAs selected in the multivariate Cox regression were used to construct PTC OS-associated signature and DFS-associated signature. The prognostic signatures were constructed as follows:

$$\mathrm{Risk score}=\sum_{i=1}^{n}\beta_{i}*Expression_{i}$$ (1)

where *n* is the number of candidate genes, *β*_*i*_ is the coefficient of gene *i* in multivariate regression analysis, and *Expression*_*i*_ is the expression level of gene *i* that has been normalized by TMM.

Based on the risk score, the PTC patients were divided into high and low-risk groups by cut-off median. Time-dependent receiver operating characteristic (ROC) and Kaplan-Meier survival curve analyses were performed by R package of “survivalROC,” “survival,” and “survminer.” Area under curve (AUC) value from the ROC curve and concordance index (C index) were calculated to determine the prognosis accuracy of the two signatures.

Using the other clinicopathological factors associated with PTC patients’ OS or DFS time as confounding variables, clinical characteristics including age, gender (male/female), and stage (I, II, III, IV) were also analyzed using univariate and multivariate Cox regression. This stratified analysis was conducted to determine whether the prognostic signature is independent of these clinical factors.

### Estimation of Tumor Microenvironment

In order to evaluate the proportions of all 22 immune cells in PTC tissues, CIBERSORT methods were used based on the gene expression profile by running CIBERSORT script from the website (http://rdrr.io/github/singha53/amritr/src/R/supportFunc_cibersort.R). The sums of immune cells of each PTC patient were equal to 1. The Wilcoxon test was used to test the prominent difference of immune cells’ proportions between high and low-risk groups that was divided according to OS-associated signature and DFS-associated signature respectively. The Pearson correlation coefficient was calculated to study the correlations between 22 immune cells and key genes involved in two risk models with the cutoff values of |Cor|>0.2 and *p* < 0.05. So, the distinctive immune cells were identified that not only show significant differences between high and low-risk groups but are correlated with the expression levels of genes. Finally, univariate and multivariate Cox regression analyses were used to further identify those which may be associated with OS or DFS of patients.

### CMap Analysis

The CMap online tool (http://broadinstitute.org/cmap) was used to predict the effect of drugs on the particular gene expression patterns in tumors. In order to study functional connections between the key genes associated with OS and DFS of PTC patients and bioactive chemicals, the up-regulated and down-regulated tags from the key genes were uploaded into the CMap online tool. How closely a compound is connected to the uploaded signature depends on the connectivity score with a range from −1 to 1. A positive connectivity score indicates that the compound promotes the query gene expression, whereas a negative connectivity score indicates that the compound represses the query gene expression.

## Results

### Identification and Validation of Hub lncRNAs for Marking PTC

**FIGURE 1:**
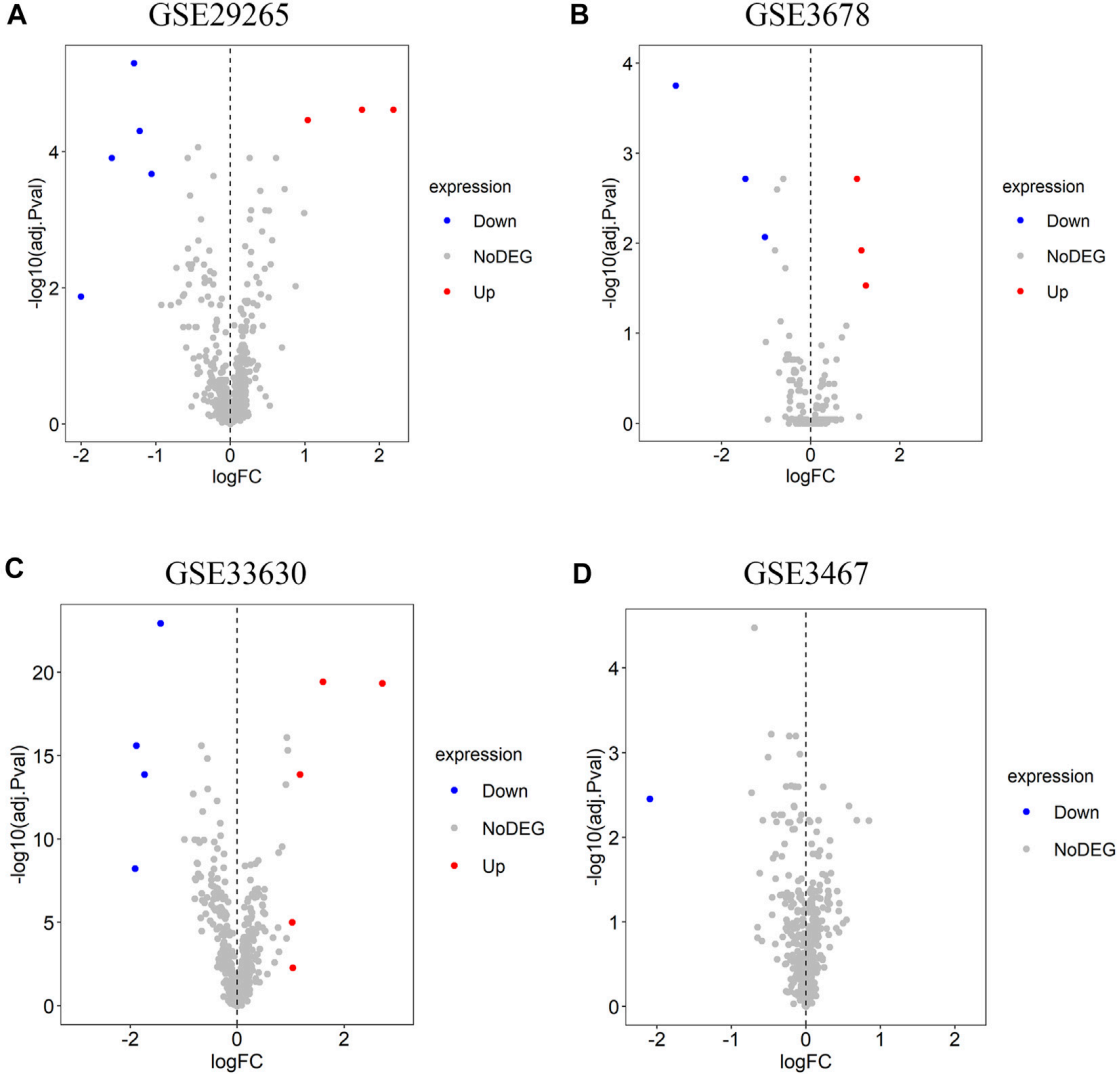
Identification of differentially expressed lncRNAs in each dataset. **(A)** GSE29265, **(B)** GSE3678, **(C)** GSE33630, **(D)** GSE3467. The red pots represent the up-regulated lncRNAs and the blue pots represent the down-regulated ones with the cutoff criteria of |logFC| > 1 and adjusted *p* < 0.05. The grey pots represent lncRNAs with no prominent expression difference.

Four lncRNA gene chips (GSE29265, GSE3678, GSE33630, and GSE3467) based on the same sequencing platform were selected in this study. In total, eight differentially expressed lncRNAs were recognized in GSE29265 gene chip, including three lncRNAs with higher expression and five lncRNAs with lower expression (Figure 1A). A total of six differentially expressed lncRNAs were identified from GSE3678 gene chip, of which three are up-regulated and thre are down-regulated lncRNAs (Figure 1B). Moreover, there are nine differential lncRNAs in GSE33630 gene chip, containing five up-regulated and four down-regulated ones (Figure 1C). Only one down-regulated lncRNA was recognized in GSE3467 (Figure 1D). So, we can see that different gene chips give different differential lncRNAs. Then we used RRA method for integration and further screening so as to achieve more distinctive hub lncRNAs. Through rank analysis by RRA method among the four expression matrices, five hub lncRNAs were identified.

**FIGURE 2:**
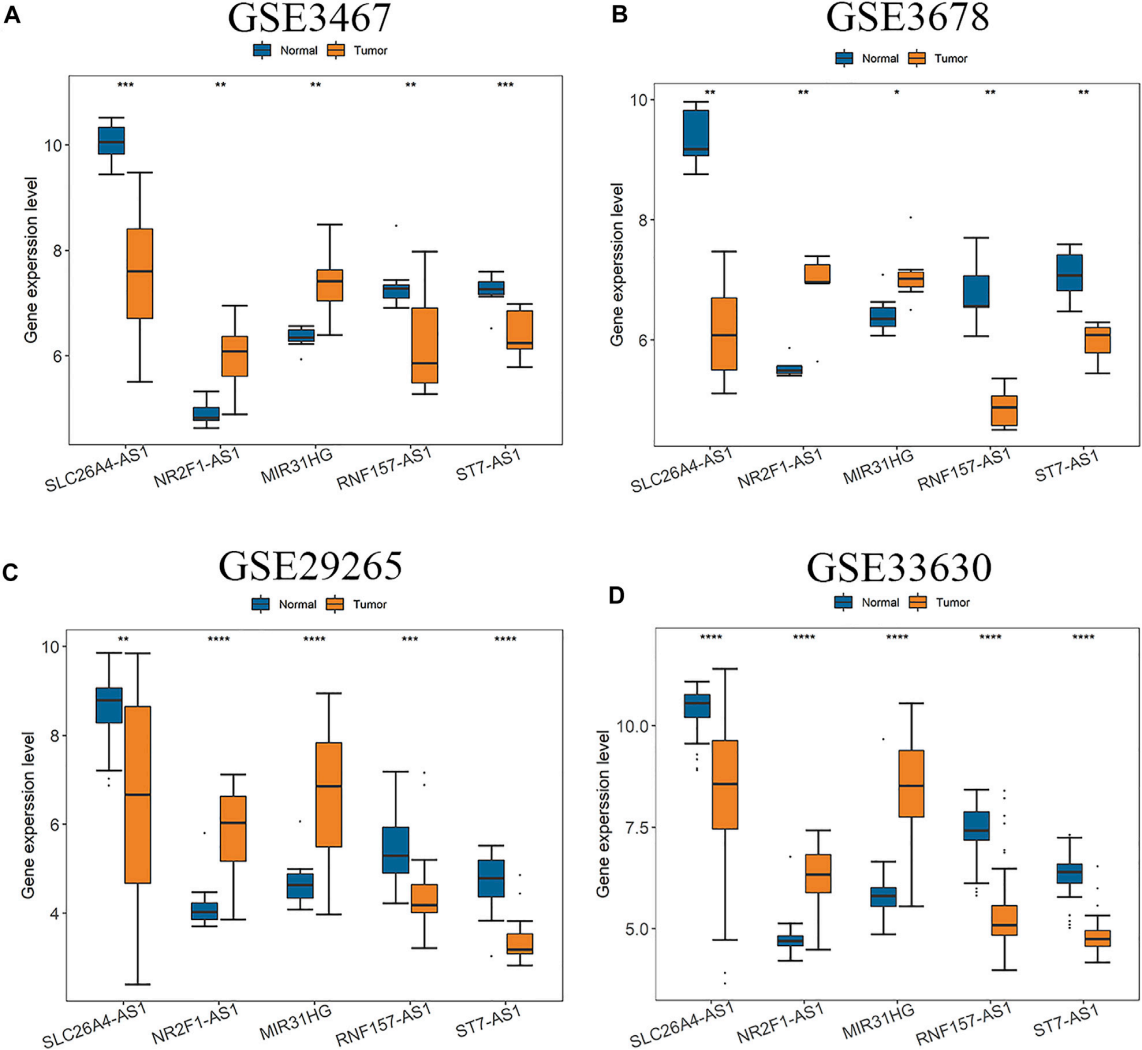
Expressions of five hub lncRNAs in PTC compared with normal tissues in **(A)** GSE3467, **(B)** GSE3678, **(C)** GSE29265 and **(D)** GSE33630 dataset. (*: *p* < 0.05, **: *p* < 0.01, ***: *p* < 0.001).

The five hub lncRNAs are SLC26A4-AS1, RNF157-AS1, NR2F1-AS1, ST7-AS1, and MIR31HG. Among them, RNA expressions of NR2F1-AS1 and MIR31HG in PTC tissues were significantly up-regulated compared with normal tissues, while expressions of the other three genes were significantly down-regulated in all four GEO datasets (Figure 2). In order to verify this observation, expression levels of these five hub genes were also analyzed based on two other validation datasets of GEPIA database and TCGA (Supplementary Figures S1, S2). Since RNF157-AS1 is not included in the GEPIA database, Supplementary Figure S1 only gives differential analysis results of four other genes. We can see that all five hub lncRNAs are differentially expressed in all datasets. NR2F1-AS1 and MIR31HG are always up-regulated and the other three genes are down-regulated in all six or five datasets.

**FIGURE 3:**
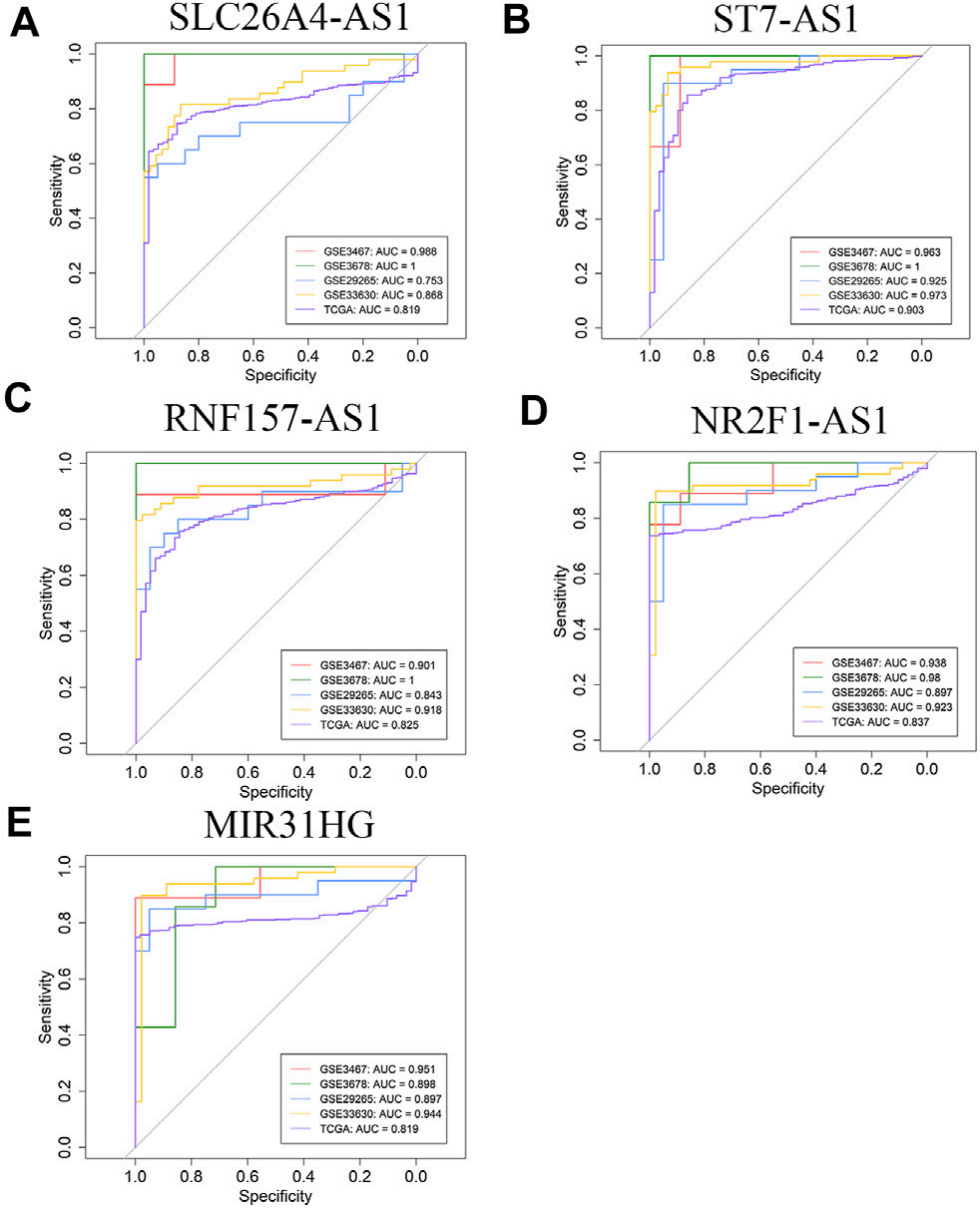
ROC curve analysis of five hub lncRNAs diagnosis in GSE3467, GSE3678, GSE29265, GSE33630, and TCGA cohort. **(A)** SLC26A4-AS1 **(B)** ST7-AS1 **(C)** RNF157-AS1 **(D)** NR2FA-AS1 **(E)** MIR31HG.

In order to further verify the potentiality of five hub lncRNAs for marking PTC, the diagnostic performance of these five hub lncRNAs were demonstrated by ROC curve analysis based on four GEO datasets and TCGA, as shown in Figures 3A–E. For each of them, the AUC value is higher than 0.90 in at least two datasets. SLC26A4-AS1 is the exception as it gives an AUC value of 0.753 in GSE29265, while all other 24 AUC values are higher than 0.80. ST7-AS1 yields the best diagnostic performance in all five databases with all five AUC values higher than 0.90 (Figure 3B) and those of SLC26A4-AS1, ST7-AS1, and RNF157-AS1 in GSE3678 are equal to 1. The results illustrate that the five hub genes screened out by us also yield excellent diagnostic efficiency between PTC and normal tissues. These validation tests suggest that the five hub lncRNAs are all reliable and potential biomarkers for marking PTC.

Finally, a deep literature-exploring was implemented and all five hub genes have been confirmed as having important roles in PTC and other cancers. The overexpression of SLC26A4-AS1 could decrease cell migration, invasion, and proliferation, and thus had anti-oncogenic effects in PTC (Wang DP. et al., 2020). But NR2F1-AS1 was reported to promote invasion and invasion of PTC (Yang et al., 2020). Besides, MIR31HG was observed to promote cell proliferation and cell cycle progression and inhibit cell apoptosis, and it could be a potential therapeutic target for head and neck squamous cell carcinoma in the study by Wang et al. (2018). As mentioned in previous research, up-regulated ST7-AS1 could expedite migration and invasion in gastric cancer and it promoted the oncogenicity of cervical cancer cells by ST7-AS1/miR-543/TPRM7 (Cai et al., 2020; Qi et al., 2020). Xu and Xu (2020) observed that the higher expression of RNF157-AS1 motivated the proliferation of ovarian cancer cells, while the overexpression of RNF157-AS1 decreased the chemoresistance; thus, ovarian cancer patients with overexpressed RNF157-AS1 have better prognosis.

### lncRNA-mRNA Co-Expression Network

**FIGURE 4:**
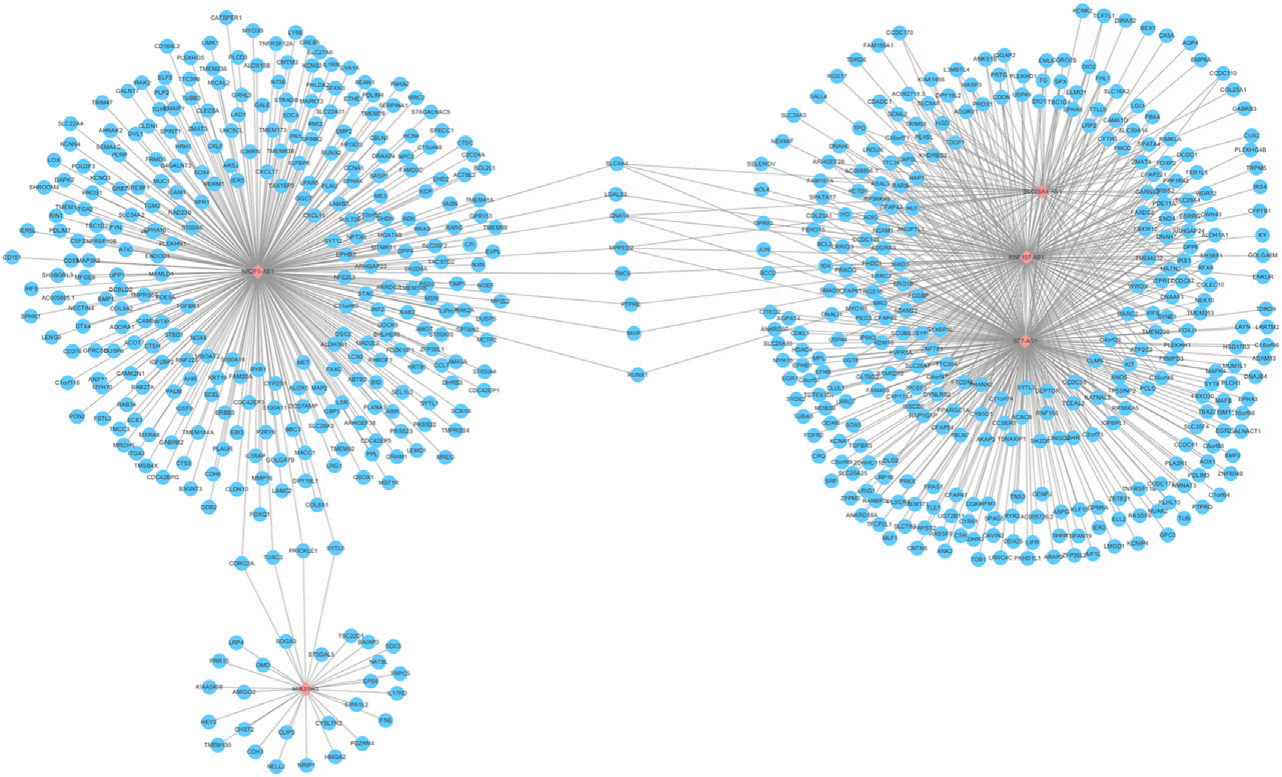
lncRNA-mRNA co-expression network. The network includes the five hub lncRNAs (pink pots) and 650 mRNAs (blue pots).

Further, functions of the co-expressed mRNAs with hub lncRNAs were investigated. By Pearson correlation analysis with the cutoff values of |Cor| > 0.5 and *p* < 0.05, the interactions between five lncRNAs and 2716 differential mRNAs in TCGA were researched. A total of 647 mRNAs were significantly related to the five hub lncRNAs, so the lncRNA-mRNA co-expression network was constructed. The network graph is shown in Figure 4. We can see that SLC26A4-AS1, RNF157-AS1, and ST7-AS1 share more interacting mRNAs, which may indicate that there are coordinated interactions among three lncRNAs by sharing common mRNAs. But NR2F1-AS1 individually has the most interacting mRNAs and MIR31HG has the least. So, we presented a further functional analysis on these co-expressed mRNAs using GO, KEGG pathway, and DO analysis.

**FIGURE 5:**
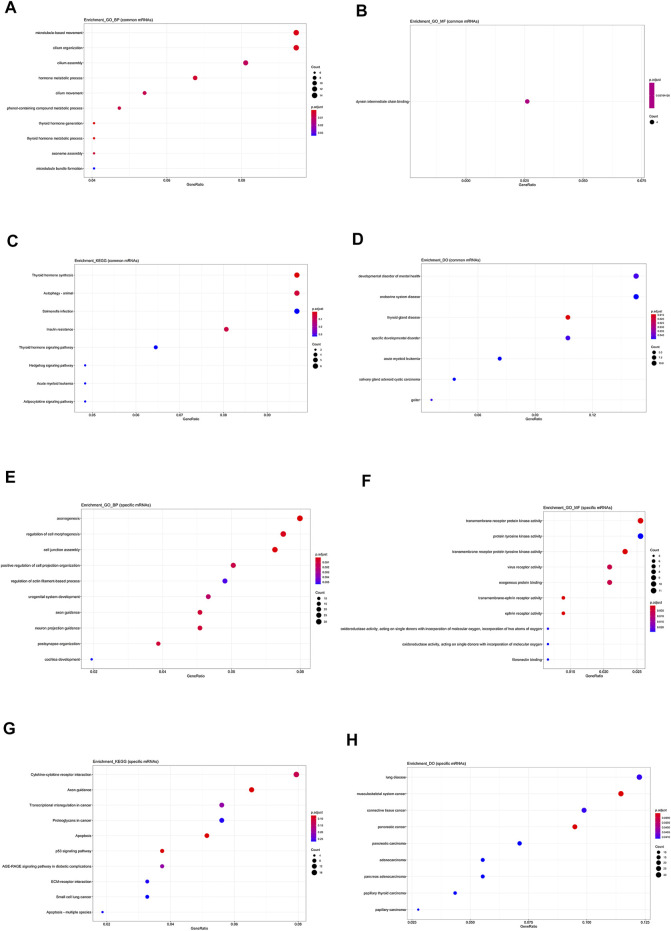
Functional analysis on common and specific mRNAs in lncRNA-mRNA co-expression network. **(A)** The biological process items of common mRNAs by GO analysis. **(B)** The molecular function items of common mRNAs by GO analysis. **(C)** Functional enrichment analysis by KEGG for common mRNAs. **(D)** Functional enrichment analysis by DO for common mRNAs. **(E)** The biological process items of specific mRNAs by GO analysis. **(F)** The molecular function items of specific mRNAs by GO analysis. **(G)** Functional enrichment analysis of KEGG for specific mRNAs. **(H)** Functional enrichment analysis of DO of specific mRNAs.

Firstly, mRNAs involved in lncRNA-mRNA co-expression network were divided in to common or specific ones. If mRNAs are related with two or more lncRNAs, they are defined as common and those that only connect with one lncRNA are specific mRNAs. As displayed in Figure 5, the common mRNAs are involved in the thyroid hormone generation (Figure 5A) and dynein intermediate chain binding function (Figure 5B). They are commonly associated with thyroid hormone synthesis (Figure 5C) and thyroid gland disease (Figure 5D). But specific mRNAs are most involved in axonogenesis (Figure 5E) and transmembrane receptor protein tyrosine kinase activity (Figure 5F). They may be related with p53 signaling pathway (Figure 5G) and papillary carcinoma (Figure 5H). So, the function analysis indicates that the co-expressed mRNAs that are common between five hub lncRNAs may have important roles in the development and progression of PTC.

### lncRNA-miRNA-mRNA ceRNA Network

To construct lncRNA-miRNA-mRNA ceRNA network, the starBase v2.0 was used to predict the target miRNAs of five hub lncRNAs and 713 were identified. Then, 17 target miRNAs were determined by intersecting 167 differentially expressed miRNAs in TCGA and 713 predicted miRNAs. Consequently, miRDB, miRTarBase, and TargetScan 7.2 were used to predict probable target mRNAs of the above 17 miRNAs and extracted the intersections from the three online analysis tools. By overlapping the predicted mRNAs to 2716 differential derived from TCGA, 68 target mRNAs that may exert critical functions in PTC were discovered.

**FIGURE 6:**
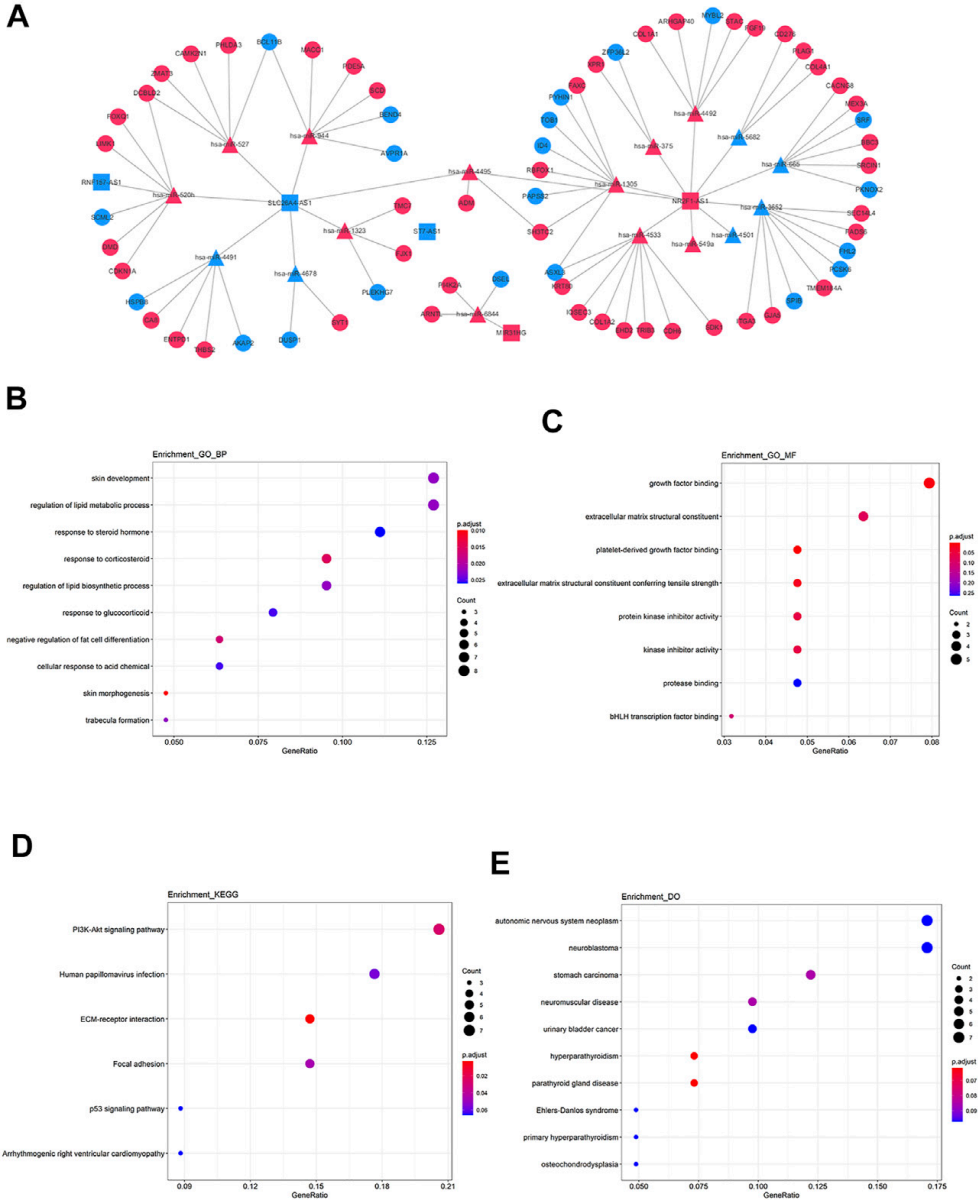
| lncRNA-miRNA-mRNA ceRNA network and functional prediction of mRNAs in network. **(A)** The network consists of three lncRNAs (rectangles), 17miRNAs (triangles), and 68 mRNAs (circles). The red pots represent up-regulated RNAs and the blue pots represent down-regulated RNAs. **(B)** The biological process items by GO analysis. **(C)** The molecular function items by GO analysis. **(D, E)** Functional enrichment analysis of DO.

Based on the achieved lncRNA-miRNA pairs and miRNA-mRNA pairs, the lncRNA-miRNA-mRNA ceRNA network was constructed (Figure 6A). The potential functional characteristics of mRNAs in this ceRNA network were also interpreted by GO, KEGG pathway, and DO analysis respectively. The 68 differential target mRNAs are enriched in BP of skin morphogenesis and respond to corticosteroid (Figure 6B) as well as MF of platelet-derived growth factor binding and extracellular matrix (Figure 6C). Previous reports have been indicated that corticosteroid could alleviate cancer-related symptoms and play an indispensable role in cancer care (Drakaki et al., 2020). In addition, lymph node metastasis is important for the treatment and prognosis of PTC patients and some platelet-derived growth factors can promote lymph node metastasis by participating in lymphangiogenesis of rectal cancer (Liu et al., 2011). The extracellular matrix can also influence cancer progression and then significantly affect the matrix composition and structure (Malandrino et al., 2018). Among the enriched pathways (Figure 6D), PI3K-Akt signaling pathway plays an extensive role in thyroid tumorigenesis and focal adhesion is also a tumor-related pathway (Hou et al., 2007; Antoniades et al., 2021). In addition, mRNAs were observably associated with hyperparathyroidism and parathyroid gland disease (Figure 6E). The above analysis could indicate to some extent that these mRNAs may play important roles in PTC.

### Construction of Prognostic Signatures and Survival Analysis

Initially, the five hub lncRNAs were used to establish the prognosis model. However, the univariate Cox analysis results of five hub lncRNAs prove that the *p*-values of five hub lncRNAs are all much higher than 0.05, as shown in Supplementary Figure S3. So, these lncRNAs were not associated with PTC patients’ OS and DFS, although they yield promising diagnostic performance.

To identify the potential RNAs with prognostic characteristics, univariate Cox proportional hazards regression analysis was performed for five lncRNAs, 17 miRNAs, and 68 mRNA expression data and those related to patient OS or DFS were selected by using *p* < 0.05 as the criteria. As a result, nine mRNAs including TMEM184A, SRCIN1, PI4K2A, FADS6, ITGA3, KRT80, ADM, TOB1, and DCBLD2 were found to be correlated with PTC DFS. On the other hand, four miRNAs and nine mRNAs, namely hsa-miR-1305, hsa-miR-4501, hsa-miR-3652, hsa-miR-665, PASS2, SCD, THBS2, ID4, FHL2, MEX3A, DSEL, DCBLD2, and TMEM184A, were significantly associated with PTC OS. Then, in order to further screen out an optimal combination from these genes, stepwise multivariate Cox regression analysis was conducted and subsequently two predictive signatures named PTC-mi1m4 (hsa-miR-1305, PAPSS2, SCD, ID4, and DCBLD2) and PTC-m3 (TMEM184A, TOB1, and FADS6) were obtained for PTC OS and DFS respectively.

For the feature genes in the prognostic risk models, their cancer-related function roles were also investigated here. A previous study by Ng et al. (2015) has shown that hsa-miR-1305 may target the genes involved in cell cycle, cell junction, and cytoskeleton. In our study the target genes are PAPSS2, SCD, and ID4 which play significant roles in various cancers. PAPSS2 is downregulated in radiation-induced PTC and has been used as a potential biomarker for radiation-induced PTC (Stein et al., 2010). ID4 is a promising target in cancer therapy and it could be involved in thyroid tumorigenesis and prevent thyroid cancer invasion and metastasis (Amaral et al., 2019). Inhibiting SCD could result in tumor cell death including anaplastic thyroid carcinoma, colorectal adenocarcinoma, renal cell carcinoma, and non-small cell lung carcinoma (von Roemeling and Copland, 2016). DCBLD2 has been reported to play a positive role in lung cancer and glioblastomas but shows a negative role in gastric and neuroendocrine cancers (He et al., 2020). For the additional three mRNAs of TMEM184A, TOB1, and FADS6 in PTC DFS model, heparin binds specifically to TMEM184A and could induce anti-proliferative signaling *in vitro* (Farwell et al., 2017). As a Tob/BTG anti-proliferation protein family member, TOB1 acts as a tumor suppressor in many cancers. Tob phosphorylation also contributes to the progression of PTC (Ito et al., 2005) and NR2F1-AS1identified as a hub gene by us could suppress proliferation of colorectal cancer cells by regulating TOB1 (Wang J. et al., 2020). FADS6 was found to be mutated in Chinese Epstein-Barr virus-positive diffuse large B-cell lymphoma (Liu et al., 2018). Overall, these genes constructing two prognostic signatures are all involved in cancer-related functions.

**FIGURE 7:**
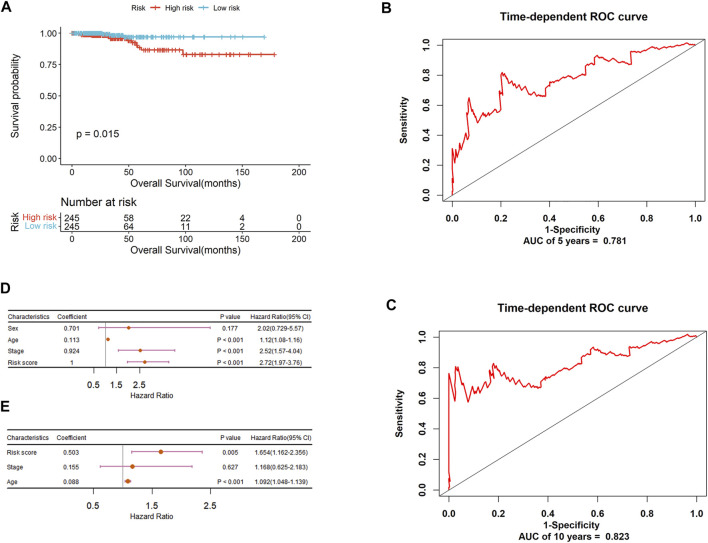
The prognostic significance of PTC-mi1m4 signature to predict OS of PTC patients. **(A)** Kaplan-Meier curve analysis for OS. **(B)** ROC validation of prognostic value of PTC-mi1m4 signature for predicting 5-years survival of PTC patients. **(C)** ROC validation of prognostic value of PTC-mi1m4 signature for predicting 10-years survival of PTC patients. **(D)** Forest plot summary of univariable analysis of sex, age, stage, and risk score. **(E)** Forest plot summary of multivariable analysis of age, stage, and risk score.

**FIGURE 8:**
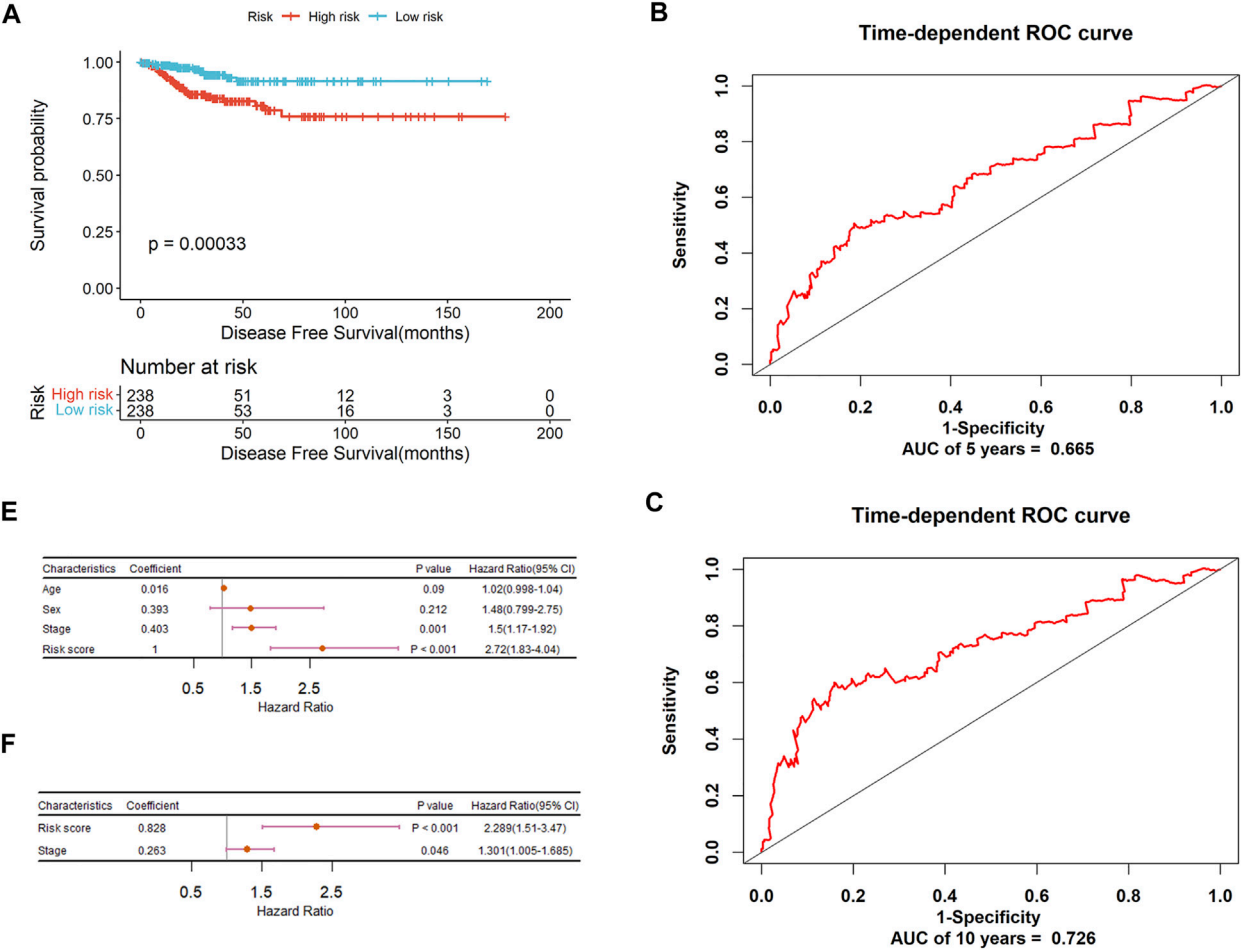
The prognostic significance of PTC-m3 signature to predict DFS of PTC patients. **(A)** Kaplan-Meier curve analysis for DFS. **(B)** ROC validation of prognostic value of PTC-m3 signature for predicting 5-years survival of PTC patients. **(C)** ROC validation of prognostic value of PTC-m3 signature for predicting 10-years survival of PTC patients. **(D)** Forest plot summary of univariable analysis of sex, age, stage, and risk score. **(E)** Forest plot summary of multivariable analysis of stage and risk score.

The risk score of each patient was calculated and all patients were divided into high and low-risk groups using the median as the cutoff. For PTC-mi1m4, it can be seen from Figure 7A that the Kaplan-Meier analysis shows that patients with low-risk score have a higher survival rate compared to those in the high-risk group (*p* = 0.015). The time-dependent ROC analysis shows that the AUC values for predicting 5-years and 10-years OS rates are 0.781 and 0.823respectively with C index of 0.775 (Figures 7B,C), suggesting that this model yields a strong prognostic ability for predicting PTC OS. Then the stratification analysis was implemented based on risk score, age, gender, and tumor stage. As shown in Figure 7D, univariate Cox regression analysis reveals that risk score, age, and stage are associated with PTC patients’ OS, but multivariate Cox regression analysis show that risk score and age are the independent prognostic indicators for PTC patients’ OS (Figure 7E). Similarly, another prognostic signature (PTC-m3) for DFS prediction could also adequately classify PTC patients into low and high-risk groups. The survival analysis demonstrates that high-risk patients have shorter survival times than low-risk patients (Figure 8A). The AUC-ROC are 0.665 and 0.726 at five and 10 years respectively with C index of 0.676 (Figures 8B,C). After performing univariate and multivariate Cox regression analysis, the result also shows that this risk score could be an independent applicable prognostic indicator for predicting PTC patients’ DFS (Figures 8D,E).

### Immune Landscape in Patients With PTC

**FIGURE 9:**
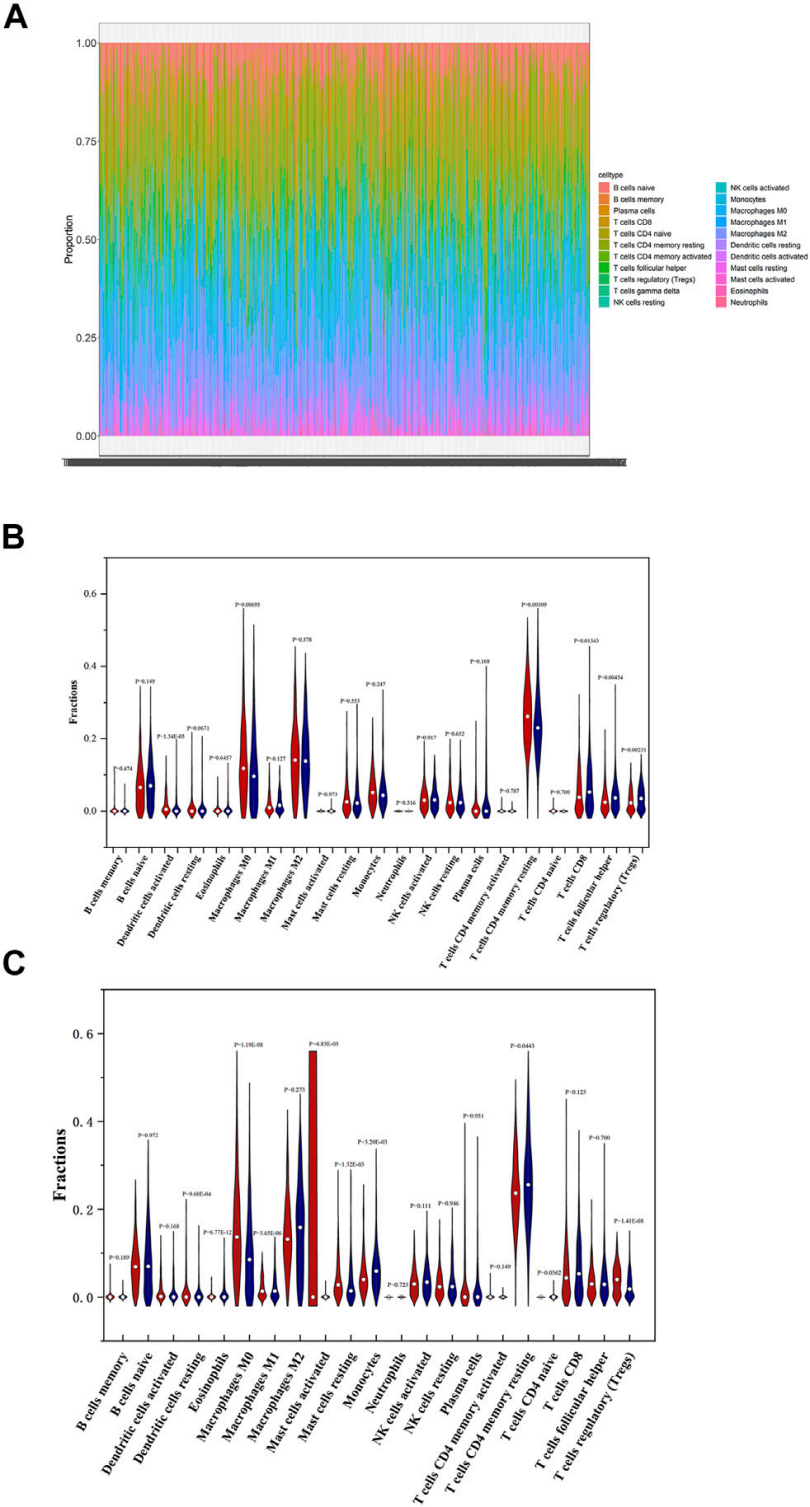
Immune landscape in low and high-risk patients with PTC. **(A)** Proportions of 22 immune cells in PTC patients. **(B)** Comparisons on the proportions of immune infiltrating cells between low and high-risk patients based on OS-associated signature. **(C)** Comparisons on the proportions of immune infiltrating cells between low and high-risk patients based on DFS-associated signature.

Understanding the tumor microenvironment (TME) is of practical significance for cancer diagnosis and treatments. The 22 immune cells form the major non-tumor constituents of tumor tissues, and can perturb the tumor signal and have an important role in cancer biology (Yoshihara et al., 2013). We know that differences in the proportion and level of tumor infiltrating immune cells may represent intrinsic characteristics of different individuals (Nie et al., 2020). In order to investigate the specific immune characteristics of PTC, the gene expression matrix of PTC dataset was used to estimate the portion of 22 immune cells by running CIBERSORT script. The proportion of immune cells in 490 PTC samples was shown in Figure 9A. We can see that the proportion of T cells CD4 memory resting is the highest, but the fraction of neutrophils is very low. It indicates that the two immune cells may play important roles in the development of PTC tumors.

Then the differences of immune cells’ proportions between high and low-risk groups divided according to OS-associated signature and DFS-associated signature were further estimated by using Wilcoxon test and displayed in Figures 9B,C, respectively. As seen in Figure 9B, compared with low-risk patients, high-risk patients have significantly higher proportions of T cells CD4 memory resting, macrophages M0, and dendritic cells activated. Lower proportions of T cells CD8, T cells follicular helper, and T cells regulatory (Tregs) are observed in high-risk patients. Pearson correlation analysis indicates that macrophages M1, macrophages M0, eosinophils, NK cells activated, dendritic cells resting, Tregs, and dendritic cells activated are associated with mRNAs that are used to construct OS-associated signature. In summary, dendritic cells activated, macrophages M0, and Tregs not only have significant differences between high and low-risk groups but are closely related with the expression levels of four feature mRNAs in OS risk model. So univariate and multivariate Cox regression analyses were also performed on three immune cells. The results in Table 1 shows that dendritic cells activated was associated with PTC OS. Moreover, it has a higher proportion in high-risk patients.

**TABLE 1:** **|** Univariate and multivariate Cox regression analyses of three immune cells and overall survival of PTC patients.

	Univariate cox regression			Multivariate cox regression		
	HR	95% CI	*p* value	HR	95% CI	*p* value
T cells regulatory (Tregs)	8.69E-05	1.52E-12-4970	0.305	0.00	0-6597.90	0.29
Mocrophages M0	30.1	0.619-1460	0.0858	121.89	1.86-7981.86	0.024
Dendritic cells activated	1.41E+07	439-4.55E+11	0.0019	3.31E+07	914.70-1.20E+12	0.0012

Figure 9C shows that the proportions of dendritic cells resting, macrophages M0, mast cells resting, and Tregs are higher and those of eosinophils, macrophages M1, mast cells activated, monocytes, and T cells CD4 memory resting are lower in high-risk patients compared to low-risk patients. Moreover, Pearson correlation analysis demonstrates that macrophages M0, eosinophils, dendritic cells activated, neutrophages, T cells CD4 naive, T cells CD8, T cells CD4 memory resting, and T cells regulatory (Tregs) are closely correlated with the three feature mRNAs in DFS-associated signature. So, macrophages M0, eosinophils, T cells CD4 memory resting, and Tregs not only have differences between high and low-risk groups but are related with the expression levels of mRNAs. Similarly, univariate and multivariate Cox regression analyses were also implemented, and Table 2 indicates that macrophages M0 is related with PTC patients’ DFS.

**TABLE 2:** **|** Univariate and multivariate Cox regression analyses of four immune cells and disease-free survival of PTC patients.

	Univariate cox regression			Multivariate cox regression		
	HR	95% CI	*p* value	HR	95% CI	*p* value
Macrophages M0	90.4	8.59-952	<0.001	102.85	8.55-1236.73	<0.001
Eosinophils	0.0188	5.75E-10-615000	0.653	19.06	0-9.00E+08	0.744
T cells regulatory (Tregs)	6.92	7.62E-04-62800	0.677	0.51	0-16667	0.898
T cells CD4 memory resting	0.434	0.0181-10.4	0.607	0.954	0.0265-34.306	0.979

In general, the proportion of macrophages M0 is higher in high-risk patients either based on OS-associated signature or DFS-associated signature, which may indicate that macrophages M0 would be unfavorable to the prognosis of PTC, since it has been demonstrated by the study of Xie et al. that macrophages M0 as well as dendritic cells activated and Tregs were observed to play a tumor-promoting role in PTC (Xie et al., 2020).

### Determination of Therapeutic Drugs by CMap Analysis

**FIGURE 10:**
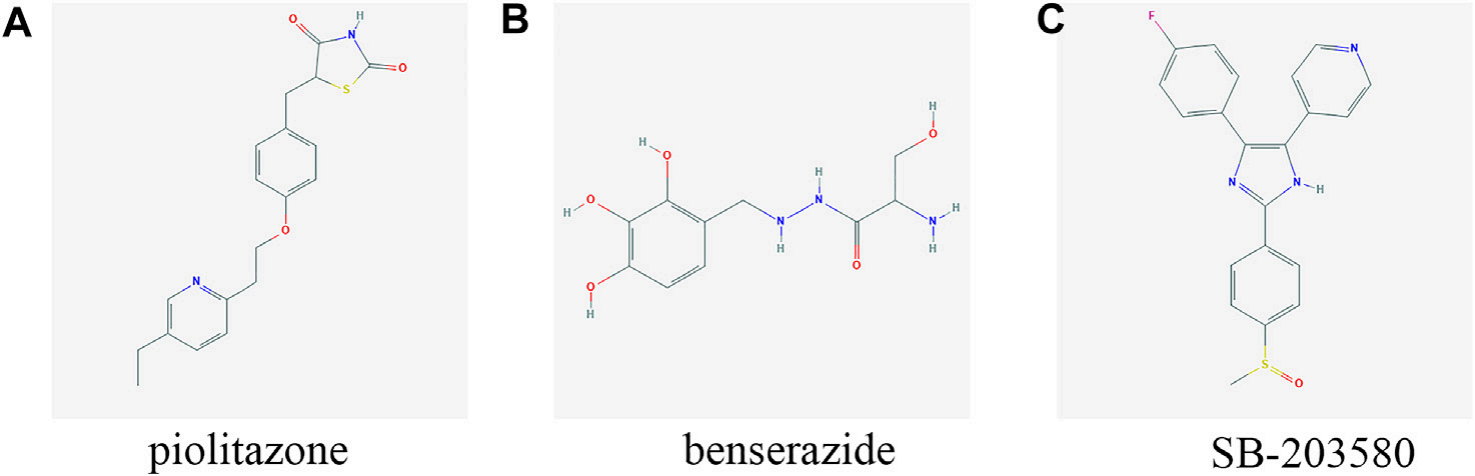
Structures of the three most significant bioactive chemicals. **(A)** pioglitazone **(B)** benserazide **(C)** SB-203580.

Discovering novel effective drugs may improve the prognosis of patients with PTC. In our two signatures, seven feature mRNAs related to the prognosis of PTC were achieved. It is expected that drugs targeted to them may be of great potential in the therapy of PTC. Except two without GPL96 probe ID, the remaining five mRNAs including PAPSS2, TOB1, ID4, SCD, and DCBLD2 were uploaded into the CMap web tool as down-regulated tags and up-regulated tags respectively to screen the compounds that can reverse the expression of these five hub genes. A negative connectivity score indicates that the compound represses the query gene expression. So, the top three bioactive compounds with connectivity scores close to -1 were determined as the potential therapeutic agents for PTC. The chemical structures of three compounds are shown in Figure 10 and the detailed information about them derived by CMap analysis are listed in Table 3.

**TABLE 3:** **|** Three bioactive compounds with the top three negative connectivity scores in the CMap analysis.

Drug	Dose, µM	Cell	Connectivity score	Up score	Down score
Pioglitazone	10	MCF7	−1	−0.935	0.772
Benserazide	14	PC3	−0.99	−0.902	0.789
SB-203580	1	MCF7	−0.97	−0.876	0.779

Among the three compounds, it has been reported that pioglitazone may be the potential drug in patients with PAX8-PPARγfusion protein (PPFP) thyroid cancer and thyroid cancer. It can also promote apoptosis in human glioblastoma LN-18 cells (Giordano et al., 2018; Ozdemir Kutbay et al., 2020; Szoka and Palka, 2020). Benserazide has been used as a drug with low toxicity for the treatment of Parkinson’s disease. It has been proven that it can suppress tumor growth by inhibiting HK2 so that it may be an antitumor agent (Li et al., 2017). SB-203580 is a p38 MAPK-specific inhibitor that could suppress IL-6-stimulated non-small cell lung cancer cells proliferation by inhibiting IL-6-induced p38 MAPK phosphorylating activity (Chang et al., 2005). Besides, p38 MAPK pathway has been reported to be activated in proliferation of PTC cells promoted by CXCL5 (Cui et al., 2019). Overall, the three compounds probably play vital roles in PTC-related biological processes and pathways, although their effects on PTC treatment remain to be explored.

## Discussion

Although the survival rate of PTC is relatively high, its recurrence rate is also high. Accurate diagnosis, prognosis for PTC patients, and discovering more potential drugs are of great significance in PTC clinical practice. lncRNAs have been indicated as an important biomarker for different cancers, such as colorectal cancer, gastric cancer, ovarian cancer, and so on (Luo and Xiang, 2021; Tan et al., 2021; Zhu and Mei, 2021). Moreover, lncRNAs could exert carcinogenic effects as ceRNAs in PTC (Zhang et al., 2019; Sui et al., 2020). In the present study, we systematically analyzed PTC-related genes and identified five hub lncRNAs for marking PTC tissues. By establishing lncRNA-miRNA-mRNA ceRNA network, two prognostic risk signatures were constructed for predicting OS and DFS of PTC respectively. Finally, three potential drugs were screened.

Firstly, five hub lncRNAs were identified by integrating four gene chips (GSE29265, GSE3678, GSE33630, and GSE3467) from GEO using differential expression analysis combined with RRA approach. The five lncRNAs of SLC26A4-AS1, NR2F1-AS1, MIR31HG, ST7-AS1, and RNF157-AS1 then underwent comprehensive validation tests. Significant expression difference could be observed between tumor and normal tissues in four GEO datasets, TCGA, and GEPIA databases, Moreover, ROC curve analysis shows that these five hub genes yield excellent diagnostic efficiency between tumor and normal tissues based on all four GEO datasets and TCGA and almost all AUC values higher than 0.8 in all five datasets. Actually, previous researchers have identified lncRNAs as prognostic markers of PTC, such as TTTY10 (Chen et al., 2019), LINC00284, RBMS3-AS1, and ZFX-AS1 (Zhao et al., 2018) and five lncRNAs of PPARG, E2F1, CCND1, JUN, and EZH2 (Sun et al., 2020) for predicting tumor recurrence in PTC. We have also performed ROC analysis for them in our four GEO datasets and TCGA. Among them, TTTY10 and LINC00284 both are included in the five datasets, but RBMS3-AS1 and ZFX-AS1 are only in TCGA. The five lncRNAs identified by Sun et al. (2020) are not in all datasets. So, the ROC analysis was performed on TTTY10, LINC00284, RBMS3-AS1, and ZFX-AS1 respectively, as shown in Supplementary Figure S4. It shows that only LINC00284 can give AUC values higher than 0.8 in three datasets and all others lower than 0.8. TTTY10 gives a poor performance with AUC values lower than 0.6.

In addition, lncRNA-mRNA co-expression network analysis shows that the common co-expressed mRNAs of the five hub lncRNAs are mainly involved in the cancer-related biological processes or pathways, which can indicate to some extent that these hub lncRNAs play crucial roles in PTC and other cancers. Finally, by deep literature-exploring, all of the five lncRNA genes have been confirmed as having important roles in cancers. All the above analysis proves that they would be potential biomarkers for PTC diagnosis.

However, the five hub lncRNA genes give poor correlation with the survival prognosis of PTC patients by univariate Cox regression analysis. So based on this, we aim to investigate the prognosis features from their interacting miRNAs and target mRNAs, since much more prognostic signatures have been constructed using miRNAs and mRNAs in cancers, such as gastric cancer, endometrial carcinoma, and so on (Cui et al., 2021; Deng et al., 2021). Among 713 target miRNAs identified by the starBase v2.0, 17 miRNAs are demonstrated to be differentially expressed in TCGA. And then miRDB, miRTarBase, and TargetScan 7.2 were used to give the reliable target mRNAs and 68 differentially expressed ones were identified in TCGA. Using five hub lncRNAs, 17 miRNAs, and 68 mRNAs, the lncRNA-miRNA-mRNA ceRNA network were constructed. Univariate and step-wise multivariate Cox regression analyses were performed and two prognostic signatures were achieved for effective prediction of PTC’s OS and DFS respectively. Here, they are named as PTC-mi1m4 and PTC-m3. The Kaplan-Meier analyses suggest that both signatures could successfully divide PTC patients into high and low-risk groups. The low-risk patients always have longer survival times than high-risk patients by two risk scores. Moreover, the time-dependent ROC analysis manifest that both of them can better predict long-term survival than short-term survival of PTC patients. The stratification analysis shows that both signatures could be independent applicable prognostic indicators of PTC even after adjusting for clinical factors such as stage, age, and gender.

The immune cells are an essential part in the tumor microenvironment and the effects of them on therapy is simulative or impedimental. Meanwhile, the activation status of immune cells may be different in different cancer tumors (Wu and Dai, 2017). Therefore, we estimated the proportions of 22 immune cells in PTC and analyzed those with significant differences between high and low-risk groups. As a result, dendritic cells activated, macrophages M0, and Tregs were demonstrated to be associated with the four feature mRNAs in OS prognostic signature. And macrophages M0, eosinophils, T cells CD4 memory resting, and Tregs were demonstrated to also be associated with the three feature mRNAs in DFS prognostic signature. The previous study by Xie et al. has displayed that all the three immune cells, including dendritic cells activated macrophages M0 and Tregs, play a tumor-promoting role in PTC (Xie et al., 2020). In our study, dendritic cells activated and macrophages M0 are associated with OS and DFS respectively by the regression analysis. Specifically, dendritic cells activated and macrophages M0 give higher proportion in high-risk patients based on OS-associated signature. So, we can speculate that they may be possible targets for immunotherapy of PTC. Tregs has an antitumor effect between PTC OS and DFS; its role may need further analysis by using wet lab experiments.

Disclosing the potential drugs that may reverse the expression of hub genes may improve the prognosis of patients with PTC. Therefore, we performed CMap analysis on the five feature mRNAs derived from two risk models to screen the potential compounds for the therapy of PTC. Three compounds (pioglitazone, benserazide, and SB-203580) were identified. Through literature-searching, all three bioactive compounds were shown to regulate PTC-related biological processed or pathways by targeting to the five feature mRNAs, but the practical applicability of those drugs should be experimentally confirmed in future researches.

## Data Availability

The original contributions presented in the study are included in the article/Supplementary Material, further inquiries can be directed to the corresponding authors.
