# Supplementary material for: Identification of Hub lncRNAs Along With lncRNA-miRNA-mRNA Network for Effective Diagnosis and Prognosis of Papillary Thyroid Cancer
Source: Front Pharmacol. 2021 Oct 13;12:748867. doi: 10.3389/fphar.2021.748867 (PMC8548639; doi:10.3389/fphar.2021.748867)
Supplement: Supplementary file 1 [file DataSheet1.DOC]

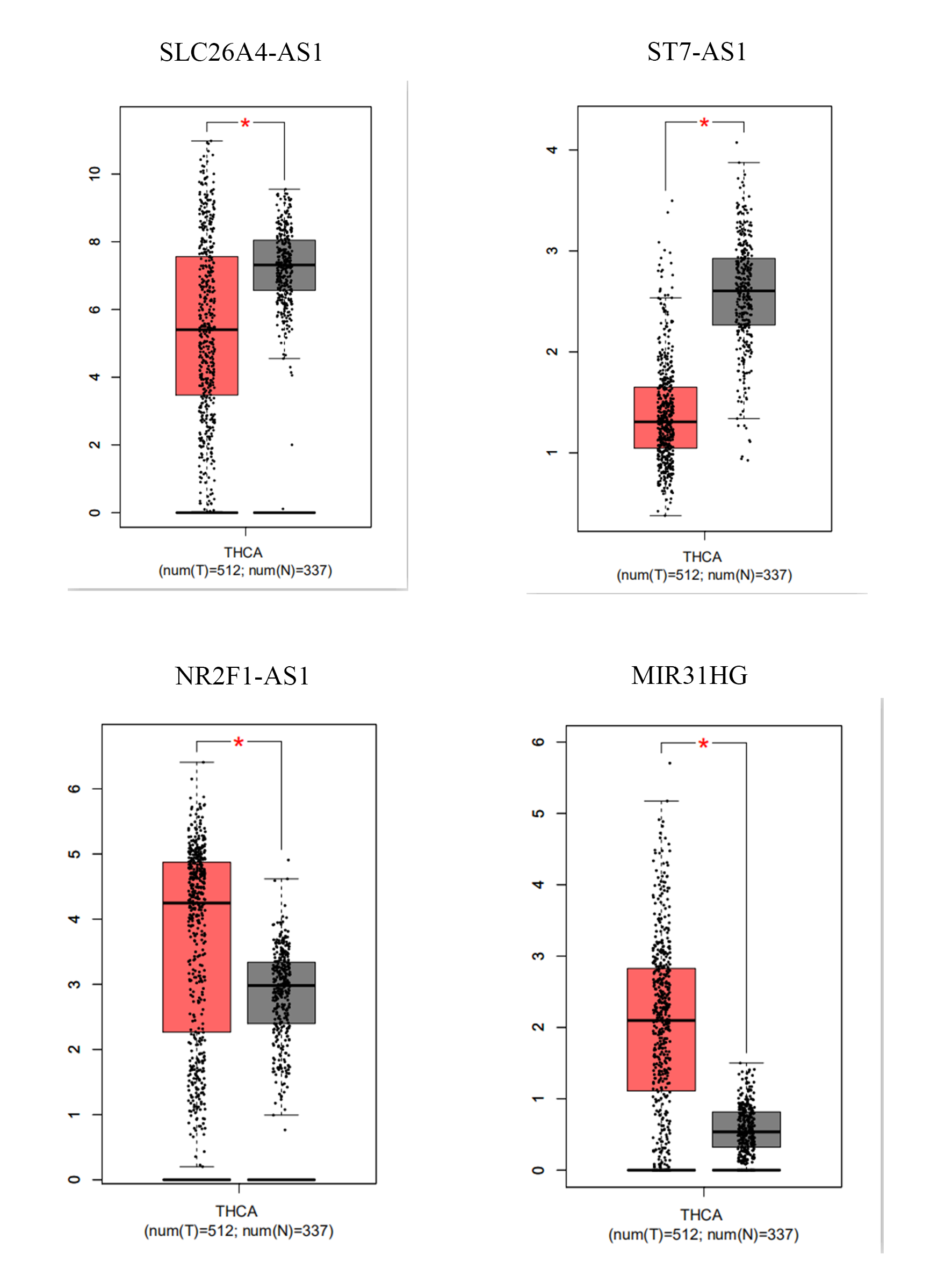


**Supplementary Figure 1.** Expressions of 4 hub lncRNAs in PTC compared with normal tissues in the GEPIA database. (*: (|log_2_FC|) ≥ 1 and p < 0.05)


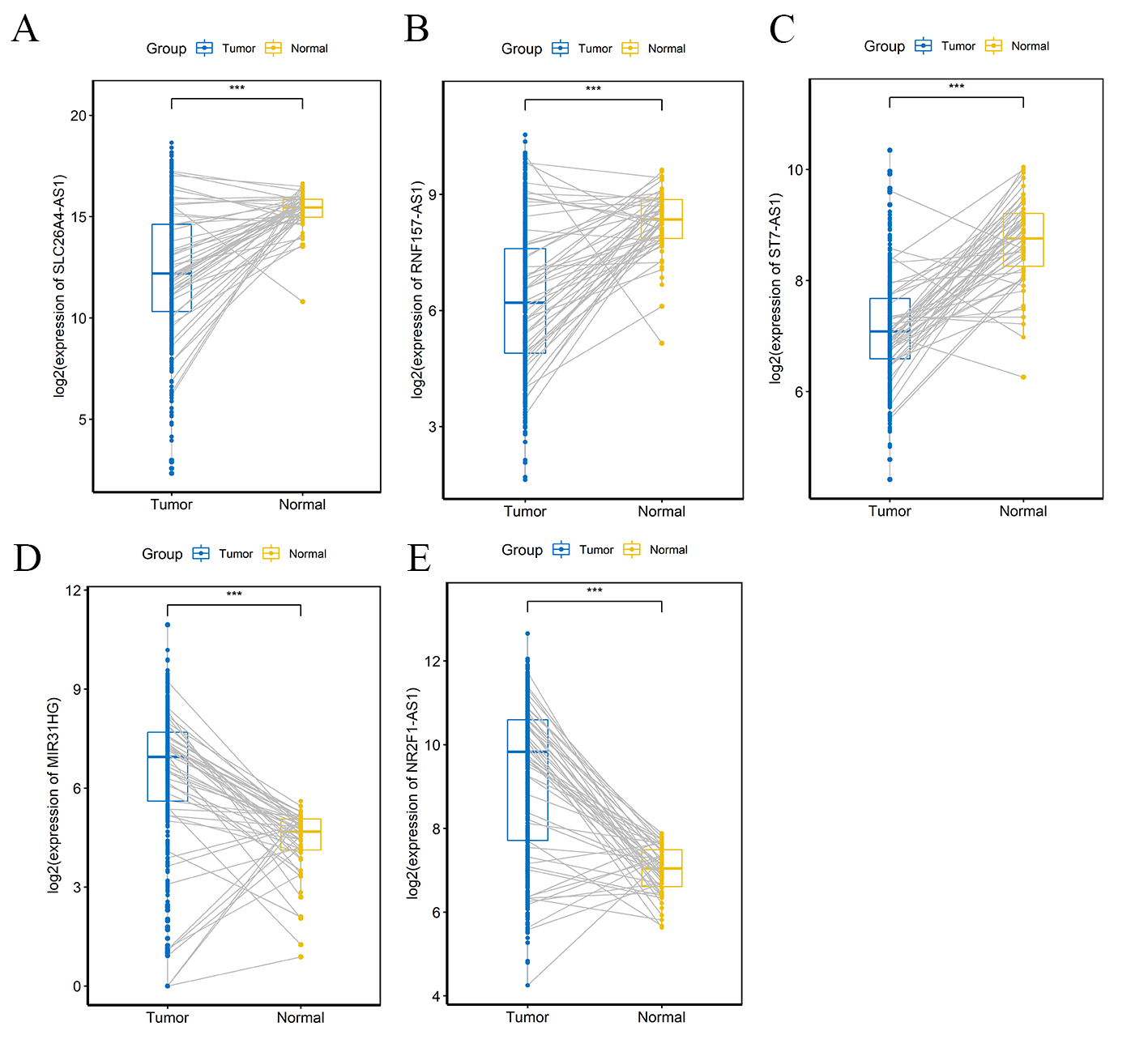


**Supplementary Figure 2.** Expressions of 5 hub lncRNAs in PTC compared with normal tissues in the TCGA database. ( ***: p < 0.001)


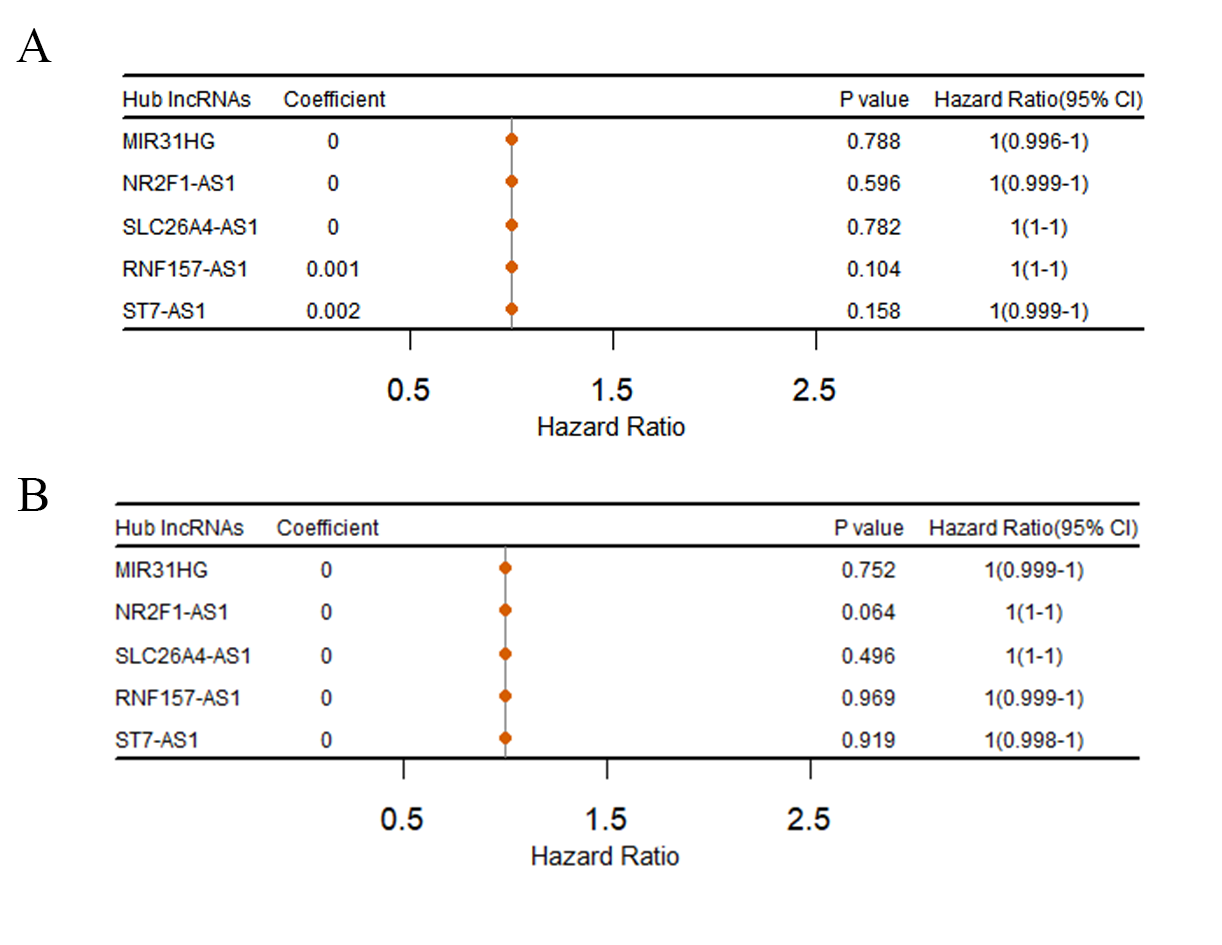


**Supplementary Figure 3.** (A) Forest plot summary of univariable analysis of 5 hub lncRNAs for PTC’s overall survival. (B) Forest plot summary of univariable analysis of 5 hub lncRNAs for PTC’s disease free survival.


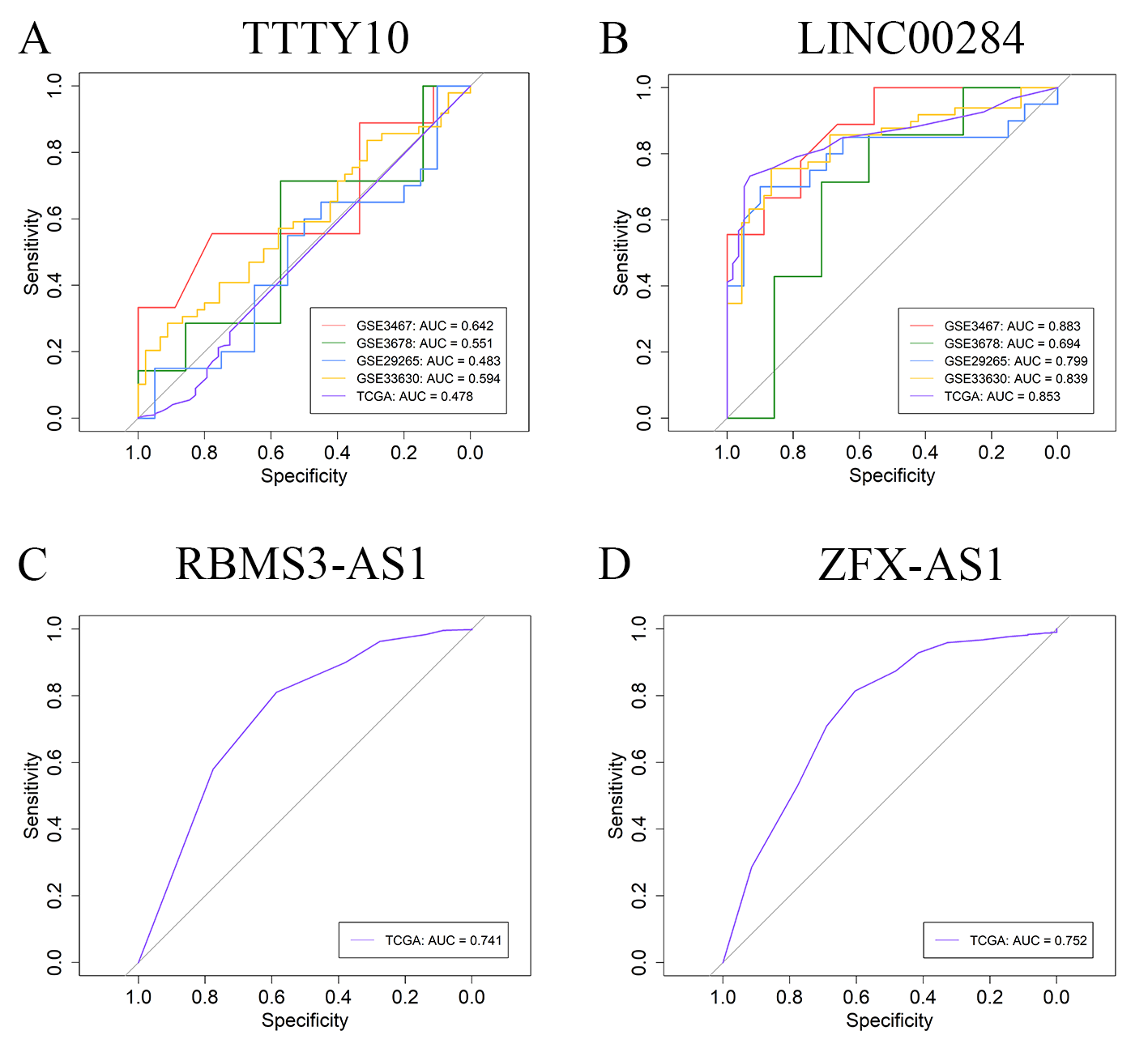


**Supplementary Figure 4.** ROC curve analysis of 4 lncRNAs diagnosis obtained from literatures in datasets that we studied. (A) TTTY10 (B) LINC00284 (C) RBMS3-AS1 (D) ZFX-AS1.
